# Fully automated detection and segmentation of intracranial aneurysms in subarachnoid hemorrhage on CTA using deep learning

**DOI:** 10.1038/s41598-020-78384-1

**Published:** 2020-12-11

**Authors:** Rahil Shahzad, Lenhard Pennig, Lukas Goertz, Frank Thiele, Christoph Kabbasch, Marc Schlamann, Boris Krischek, David Maintz, Michael Perkuhn, Jan Borggrefe

**Affiliations:** 1 Institute for Diagnostic and Interventional Radiology, Faculty of Medicine and University Hospital Cologne, University of Cologne, Cologne, Germany; 2 Philips GmbH Innovative Technologies, Aachen, Germany; 3 Department of General Neurosurgery, Center for Neurosurgery, Faculty of Medicine and University Hospital, University of Cologne, Cologne, Germany; 4 Department of Neurosurgery, Hôpitaux Robert Schuman, Luxembourg, Luxembourg; 5 Department of Radiology, Neuroradiology and Nuclear Medicine, Johannes Wesling University Hospital, Ruhr University Bochum, Bochum, Germany

**Keywords:** Image processing, Machine learning, Brain, Brain imaging, Biomedical engineering

## Abstract

In aneurysmal subarachnoid hemorrhage (aSAH), accurate diagnosis of aneurysm is essential for subsequent treatment to prevent rebleeding. However, aneurysm detection proves to be challenging and time-consuming. The purpose of this study was to develop and evaluate a deep learning model (DLM) to automatically detect and segment aneurysms in patients with aSAH on computed tomography angiography. In this retrospective single-center study, three different DLMs were trained on 68 patients with 79 aneurysms treated for aSAH (2016–2017) using five-fold-cross-validation. Their outputs were combined to a single DLM via ensemble-learning. The DLM was evaluated on an independent test set consisting of 185 patients with 215 aneurysms (2010–2015). Independent manual segmentations of aneurysms in a 3D voxel-wise manner by two readers (neurosurgeon, radiologist) provided the reference standard. For aneurysms > 30 mm^3^ (mean diameter of ~ 4 mm) on the test set, the DLM provided a detection sensitivity of 87% with false positives (FPs)/scan of 0.42. Automatic segmentations achieved a median dice similarity coefficient (DSC) of 0.80 compared to the reference standard. Aneurysm location (anterior vs. posterior circulation; P = .07) and bleeding severity (Fisher grade ≤ 3 vs. 4; P = .33) did not impede detection sensitivity or segmentation performance. For aneurysms > 100 mm^3^ (mean diameter of ~ 6 mm), a sensitivity of 96% with DSC of 0.87 and FPs/scan of 0.14 were obtained. In the present study, we demonstrate that the proposed DLM detects and segments aneurysms > 30 mm^3^ in patients with aSAH with high sensitivity independent of cerebral circulation and bleeding severity while producing FP findings of less than one per scan. Hence, the DLM can potentially assist treating physicians in aSAH by providing automated detection and segmentations of aneurysms.

## Introduction

Aneurysmal subarachnoid hemorrhage (aSAH) is caused by spontaneous rupture of an intracranial aneurysm and represents a severe neurological condition with mortality ranging between 8 and 67%^1,2^. In patients with non-traumatic SAH, accurate and reliable diagnosis of aneurysm is essential for subsequent treatment to prevent re-bleeding and further neurological deterioration^3,4^. Usually, CT-angiography (CTA) is performed immediately upon radiological proof of SAH with sensitivity rates for detection of aneurysms ranging between 85–98% compared to digital subtraction angiography (DSA), which is considered as the gold standard for aneurysm imaging^5,6^. Due to advances in imaging and diagnostic quality over the last decades as well as its non-invasiveness, CTA has the potential to replace DSA in pre-treatment assessment of aSAH in selected patients^7,8^. Timely aneurysm occlusion by endovascular or surgical means represents a key concept in modern aSAH management with selection of most suitable treatment depending on various factors such as aneurysm localization, size, and shape^3,4,9^.

With the introduction and advancements of convolutional neural networks (CNN) over the last decade, deep learning algorithms have shown great potential in performing diagnostic and analyzing tasks on medical imaging in different subspecialties^10–12^. Aneurysm detection on CTA, especially for smaller ones, proves to be challenging and misdiagnosis of aSAH can be associated with a poor clinical outcome^13–15^. Hence, the development of a deep learning model (DLM) to automatically detect and segment intracranial aneurysms would be of valuable assistance to the radiologist. This is of particular interest due to the growing workload and consequent fatigue of radiologists, which correlates with increased risk to miss relevant findings^16^.

Previous studies have proposed several approaches for (semi-) automated detection of intracranial aneurysms on CTA^10^ and time-of-flight magnetic resonance angiography (TOF-MRA)^17,18^. However, these studies focused on unruptured intracranial aneurysms (UIAs) and did not include patients with aSAH. Hence, it remains unclear, how DLM algorithms perform on patients with acutely ruptured intracranial aneurysms (RIAs) and whether the extent of hemorrhage impedes detection sensitivity.

The objective of this study was to develop and validate a DLM for automatic detection and segmentation of aneurysms on CTA in aSAH. Furthermore, we evaluated the performance of the algorithm with regard to aneurysm size, location, and bleeding severity using an independent test set of patients with aSAH.

## Materials and methods

### Patient population

**Figure 1:**
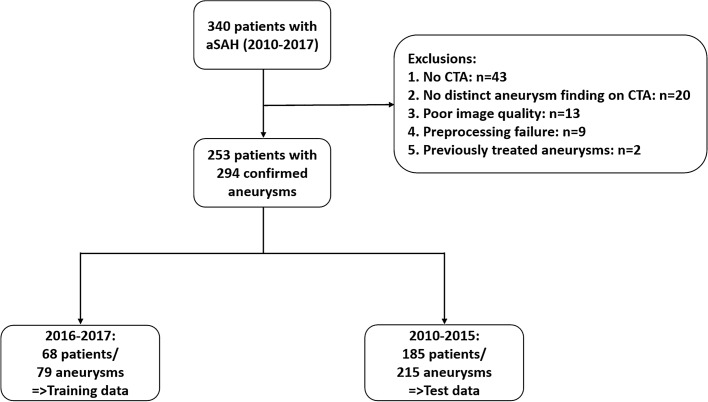
Flow chart for patient selection and inclusion scheme. aSAH = aneurysmal subarachnoid hemorrhage, CTA = CT-angiography.

All consecutive patients treated for aSAH at our tertiary-care university hospital setting between January 2010 and December 2017 were reviewed and served as our dataset (n = 340). Exclusion criteria were: (1) unavailable CTA scans, (2) no distinct aneurysm finding in CTA, (3) severe motion artifacts on CTA, (4) insufficient contrast of CTA, and (5) previously treated aneurysms. There were no exclusions due to aneurysm size. Consequently, 87 patients were excluded, resulting in 253 patients (mean age: 54.7 ± 13.9 years, 67.6% female) with 294 aneurysms for analysis. All included scans between 2016 and 2017 (n = 68 patients/79 aneurysms) served as the training dataset, whereas scans before 2016 were allocated to the independent test set (n = 185 patients/215 aneurysms). Figure 1 provides an overview of patient selection. CTA source images were anonymized and exported to IntelliSpace Discovery (ISD*, v3.0, Philips Healthcare, Best, the Netherlands*).

### Imaging

Included examinations were performed on different multidetector-CTs, namely iCT (n = 229), Brilliance 64 (n = 8), and Brilliance 16 (n = 14) (*Philips Healthcare, Best, the Netherlands*)*.* On all of these scanners, our institutional standard clinical protocol for head and neck (n = 222) or head (n = 29) CTA was used with slice thickness ranging between 0.62 to 1.25 mm. Two CTA datasets of the head from referring hospitals were included. These were acquired using Siemens Somatom Definition AS (*Siemens Healthineers, Erlangen, Germany*) and Toshiba Aquilion 64 (*Canon Medical Systems Corporation, Otawara, Japan*). (Pre-) interventional DSA was performed on a biplane angiography system (AlluraClarity FD 20/15 or FD 20 C-arm system, *Philips Healthcare, Best, the Netherlands*). Two-hundred-thirty-four patients received (pre-) interventional DSA.

### Reference standard

To establish the aneurysm count and location, aneurysms were confirmed by a neurosurgeon with four years, a radiologist with three years, and a board certified neuroradiologist with twelve years of experience in neurovascular imaging. Together, they conducted a review of the original radiology report of the CTA and double reviewed the CTA as well as DSA images (if available). Further, they collectively reviewed non-enhanced CT scans to determine respective Fisher grade of aSAH in consensus. Reference standard for aneurysm segmentations were provided by the above-mentioned neurosurgeon and radiologist, who performed semi-automatic 3D voxel-wise segmentations of aneurysms on ISD in consensus.

### Image preprocessing

**Figure 2:**
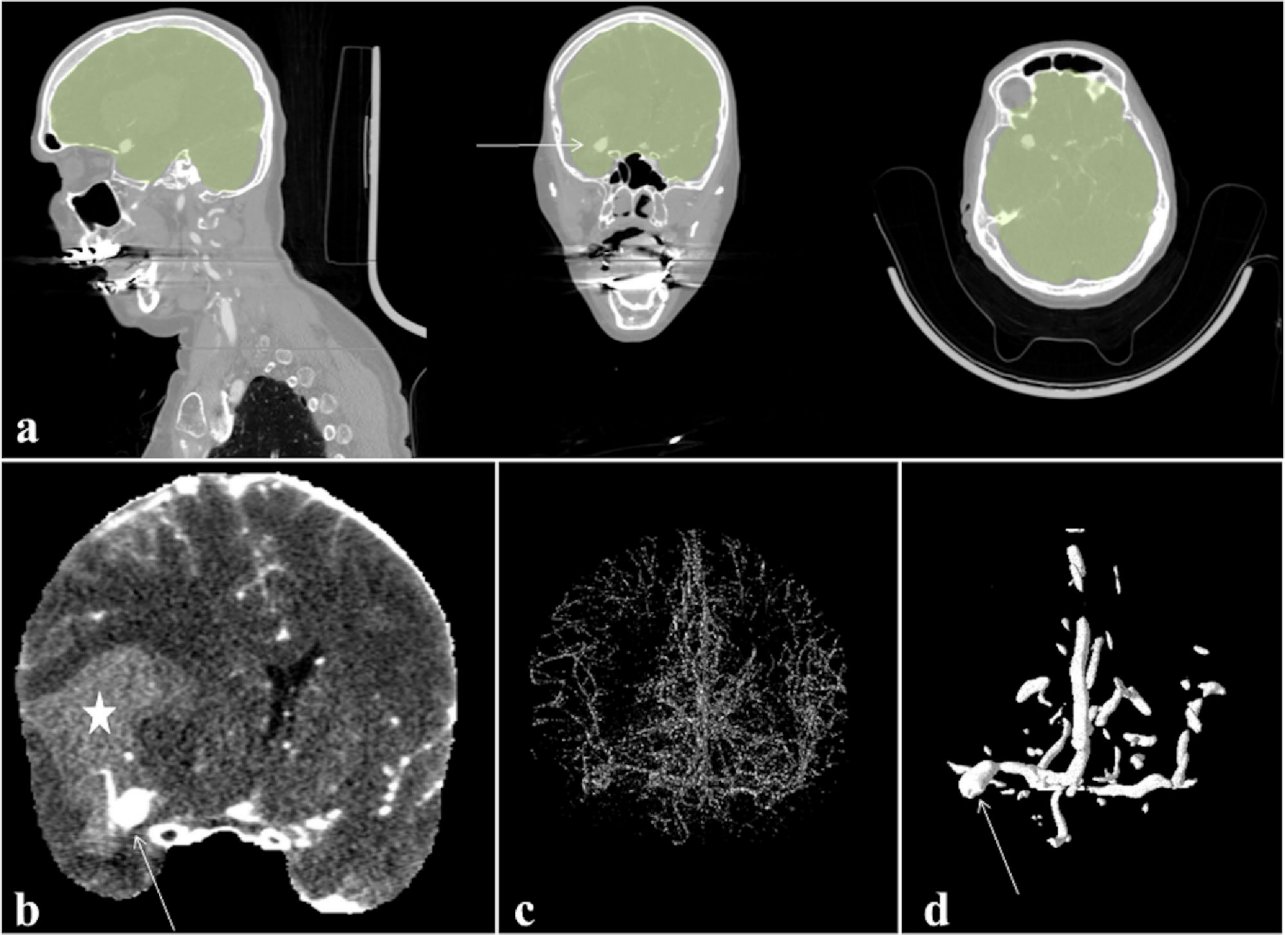
Image preprocessing workflow in a patient with an aneurysm of the right middle cerebral artery (arrow) and Fisher 4 bleeding (intraventricular hemorrhage indicated by *) with mid-line shift. Fully automated brain mask computation, overlaid in green, from acquired head and neck CTA source images (**a**). Extracted brain image (**b**). 3D rendered computed vessel enhanced images with scale 0.5–5 voxels (**c**) and 5–15 voxels (**d**).

To enable DLM-based automatic aneurysm segmentation workflow, a number of preprocessing steps were involved. First, a brain extraction algorithm was developed to compute the brain mask, using Statistical Parametric Mapping software package version 8 (SPM8; *Wellcome Trust Centre for Neuroimaging*)^19^. Second, a multi-scale vessel enhancement filter was applied to the brain masked images to enhance the arteries from the background of CTA scans^20^. In this context, two vessel-enhanced images were computed; one with scale 0.5–5 voxels and the other with scale 5–15 voxels. The two vessel-enhanced images together would help to distinguish between blood vessels and aneurysms. Third, image standardization was performed by resampling to isotropic resolution of 0.5 × 0.5 × 0.5 mm and intensity normalized. The original CTA image was normalized between 5–95% of its intensity values and the vessel enhanced images were Z-score normalized. The fully automatic image pre-processing workflow is shown in Fig. 2.

### Deep learning model development

In this study, a 3D CNN was used. The CNN is based on DeepMedic (*Biomedical Image Analysis Group, Department of Computing, Imperial College London*) consisting of a deep 3D CNN architecture with 2 identical pathways that apply different image resolutions to capture contextual information^21^. 3D image-segments centered at the same image location provide inputs to the two pathways. However, for the second pathway, the image is down-sampled to a third of its original size. The model comprises of 11 layers with kernels of size 3^3^. The model also consists of residual connections for layers 4, 6, 8, and 10 whereas layers 9 and 10 are fully connected.

A number of preliminary experiments were carried out to define the model training strategy. Based on this work, we found it most promising to use three different training procedures, henceforth referred to as DLM-Orig, DLM-Vess and DLM-LDim.

(I) For DLM-Orig, the input to the CNN is a single channel original CTA image with the size of extracted image-segments for training being set to 25^3^ voxels. (II) For DLM-Vess, the input to the CNN is multi-channel comprising of the CTA source images and the two vessel enhanced images. The size of the extracted image-segments was set to 25^3^ voxels. (III) For DLM-LDim, CTA source images represent the input, but the size of the extracted image-segments was increased from 25^3^ to 45^3^ voxels.

To increase the amount of training samples for all three models, image augmentation was employed by flipping the images along their axes. Training batch size was set to 15, batch normalization was applied and parametric rectified linear unit was used as the activation function, Dice similarity coefficient (DSC) was provided as the loss function and the number of training epochs was set to 30.

The three DLMs were trained on the training-set by a five-fold-cross-validation approach using an 80–20% training-validation split without overlapping data. Similar to the work of Kamnitsas et al.^22^, we created an ensemble model by combining the outputs of the three separate DLMs. We refer to this combination strategy as DLM-Ens.

During inference, the trained DLMs (DLM-Orig, DLM-Vess and DLM-LDim) were applied to the test dataset. Each trained DLM consisted of five individual sub-models from the five-fold-cross-validation training approach. Outputs from these five sub-models were fused together using simultaneous truth and performance level estimation (STAPLE)^23^. Subsequently, STAPLE outputs from the three DLMs were passed to DLM-Ens to produce final aneurysm segmentation prediction.

### Statistical analysis

Statistical analysis was performed with SPSS (V22.0; *IBM Armonk, NY, USA*), with P < .05 considered statistically significant. Categorical variables (e.g. scale results) are presented in form of percentages, frequencies, and medians. Comparison of paired nonparametric variables was performed with Wilcoxon signed-rank tests. Normally distributed variables are given as mean ± standard deviation with comparisons being conducted using Student’s t-test.

Following measures were computed in order to determine the detection sensitivity of the aneurysms with TP being true positives, FP false positives and FN false negatives:

$$Sensitivity=Recall=\frac{TP}{TP+FN}*100$$

$$Precision=Positive Predictive Value=\frac{TP}{TP+FP}*100$$

$$F1 score=2*\frac{\left(Sensitivity*Precision\right)}{\left(Sensitivity+Precision\right)}/ 100$$

Since no CTA scans without aneurysms were included, a true specificity cannot be determined; hence, precision was calculated, as usually conducted in machine learning tasks. To evaluate the segmentation performance of the DLMs, the automatically obtained segmentations ${(S}_{DLM})$ were compared to the manual annotations $({S}_{RS})$ with spatial overlap measure between the segmentations being computed using DSC:

$$DSC({S}_{RS}, {S}_{DLM})=\frac{2|{S}_{RS} \cap {S}_{DLM}|}{|{S}_{RS}|+|{S}_{DLM}|}$$

Resulting DSCs are reported as median. For quantitative volumetric measurements, Pearson’s correlation coefficient (r) was calculated.

### Ethics approval and consent to participate

The local institutional review board approved this retrospective, single-center study (reference number: 19-1329; Ethikkomission der Medizinischen Fakultät der Universität zu Köln) and waived the requirement for written informed patient consent. All methods were conducted in accordance with the relevant guidelines and regulations.

## Results

Baseline patient and aneurysm characteristics were comparable between the training and validation groups, as outlined in Table 1.

**Table 1:** Patient and aneurysm characteristics of training and test sets. WFNS = World Federation of Neurosurgical Societies. SD = standard deviation.

Characteristic	Training set (n = 68)	Test set (n = 185)	P value
**Patient age** (years; mean ± SD)	53.0 ± 12.8	55.1 ± 13.8	.31
**Gender**			.75
Female	47 (69.1%)	124 (67%)	
Male	21 (30.9%)	61 (33%)	
**WFNS grade**			.46
1	21 (30.9%)	54 (29.2%)	
2	12 (17.6%)	23 (12.4%)	
3	6 (8.8%)	27 (14.6%)	
4	6 (8.8%)	26 (14.1%)	
5	23 (33.8%)	55 (29.7%)	
**Fisher grade**			.18
1	2 (2.9%)	0 (0%)	
2	3 (4.4%)	13 (7%)	
3	24 (35.3%)	83 (44.9%)	
4	39 (57.4%)	89 (48.1%)	
**Aneurysm volume** (mm^3^; mean ± SD)	187.1 ± 296.3	145.6 ± 223.5	.26
**Aneurysm location**			.64
Internal carotid artery	20 (25.3%)	46 (21.4%)	
Anterior cerebral artery	24 (30.4%)	80 (37.2%)	
Middle cerebral artery	24 (30.4%)	66 (30.7%)	
Posterior circulation	11 (13.9%)	23 (10.7%)	

### Evaluation of the different DLMs on the training data

In the training set, 79 aneurysms were identified as the reference standard.

Using five-fold-cross-validation for each of the three DLMs (DLM-Orig, DLM-Vess, and DLM-LDim), an overall sensitivity of 76%, 76%, and 70%, a DSC of 0.54, 0.58, and 0.69, and average false positives (FPs)/scan of 2.10, 2.00, and 0.71 were observed.

**Figure 3:**
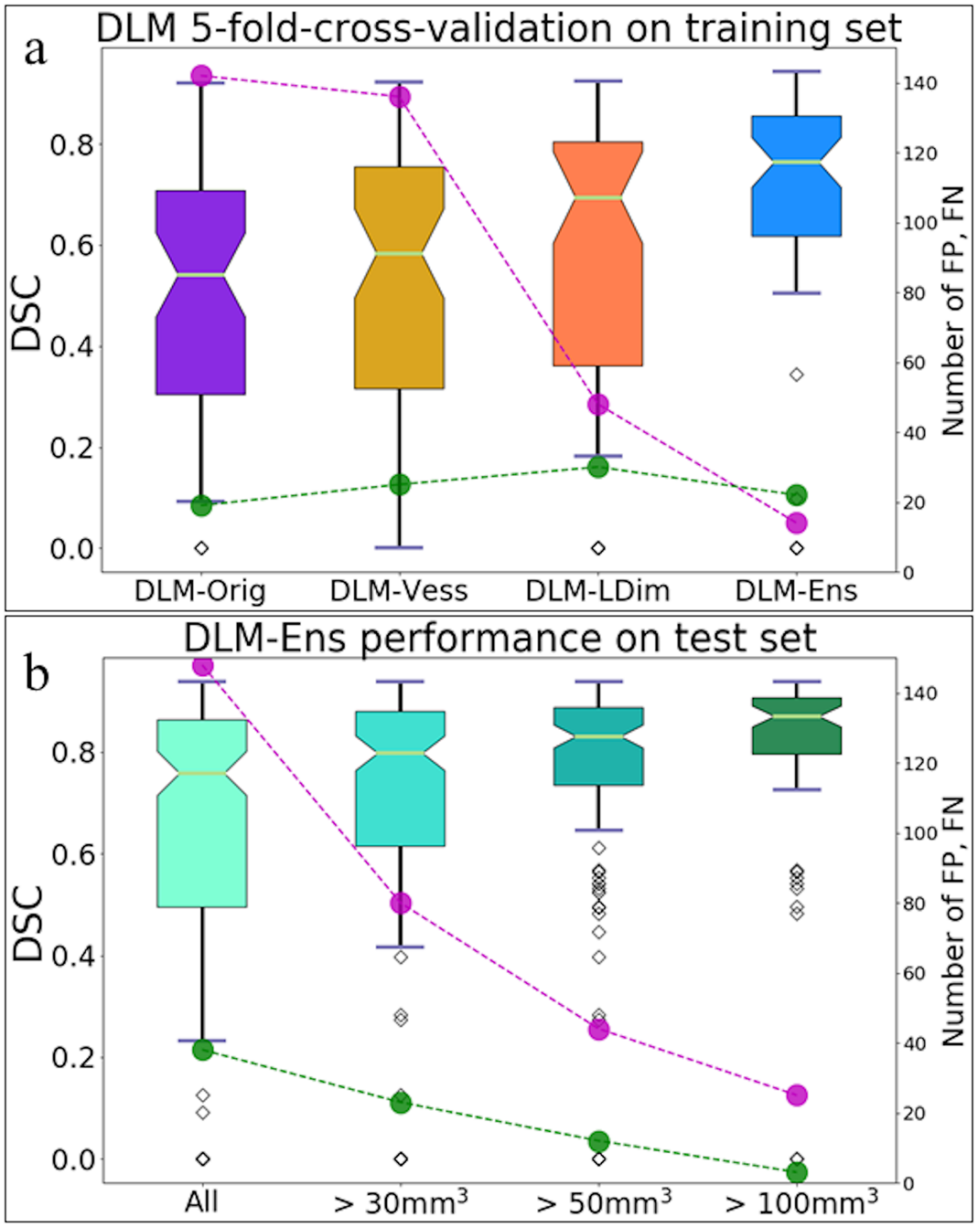
Performance of the different DLMs on the training set using five-fold-cross-validation (**a**). Segmentation performance of the DLM-Ens on the independent test set with respect to aneurysm volumes (**b**). Magenta circles represent the total number of false positives and green circles indicate the total number of false negatives.

In contrast, the DLM-Ens provided a sensitivity of 72% with median DSC of 0.74, precision of 80% and FPs/scan of 0.21. For aneurysms > 30 mm^3^ (n = 64) and > 50 mm^3^ (n = 55), the DLM-Ens achieved higher sensitivity (84%, 94%) and higher DSC (0.79, 0.81) with high precision (88%, 94%) and decreased number of FPs/scan (0.1, 0.05). In this context, Table 2 and Fig. 3a provide detailed results of the different DLMs.

**Table 2:** Sensitivity, median dice similarity coefficient (DSC), precision, F1 score, and average false positive (FPs)/scan for five-fold-cross-validation results of the different DLMs as well as results for DLM-Ens in relation to aneurysm volume.

	DLM-Orig	DLM-Vess	DLM-LDim	DLM-Ens	DLM-Ens > 30 mm^3^	DLM-Ens > 50 mm^3^
Sensitivity (detected/missed)	76% (60/19)	76% (60/19)	70% (55/24)	72% (57/22)	*84% (54/10)*	*91%* *(50/5)*
DSC (median)	0.54	0.58	0.69	0.74	*0.79*	*0.81*
Precision (detected/FP)	30% (60/142)	31% (60/136)	53% (55/48)	80% (57/14)	*88%* *(54/7)*	*94%* *(50/3)*
F1 score	0.43	0.44	0.59	0.76	*0.86*	*0.93*
FPs/scan (FP/number of scans)	2.10 (142/68)	2.00 (136/68)	0.71 (48/68)	0.21 (14/68)	*0.10* *(7/68)*	*0.05* *(3/68)*

### Evaluation of the DLM-Ens on the test cohort

Based on manual 3D segmentations, 215 aneurysms with a mean volume of 145.8 ± 230.3 mm^3^ were identified as the reference standard on the independent test set.

#### Overall volumetric correlation with the reference standard

For true positive aneurysm findings, the DLM achieved a mean volume of 154.4 ± 236.0 mm^3^, thus achieving a significant correlation with manual segmentations (r = 0.95, P < .001). The strongest correlation between manual annotations and automatic DLM segmentations was observed for large aneurysms, while correlation was insignificant (P = .810) for small aneurysms, as detailed in Table 3 and Fig. 3b.

**Table 3:** Overall correlation between manual reference standard (RS) and DLM-Ens in the combined test cohort in relation to aneurysm volume using Pearson correlation (r).

	RS	DLM-Ens	r	P value
**Volume**				
overall (mm^3^; mean ± SD)	145.8 ± 230.3	154.4 ± 236.0	0.95	< .001
< 30 mm^3^ (mm^3^; mean ± SD)	18.7 ± 7.0	38.4 ± 25.7	-0.05	.810
30–100 mm^3^ (mm^3^; mean ± SD)	54.5 ± 17.9	49.7 ± 32.8	0.46	< .001
> 100 mm^3^ (mm^3^; mean ± SD)	345.8 ± 309.9	317.3 ± 308.2	0.94	< .001

**Figure 4:**
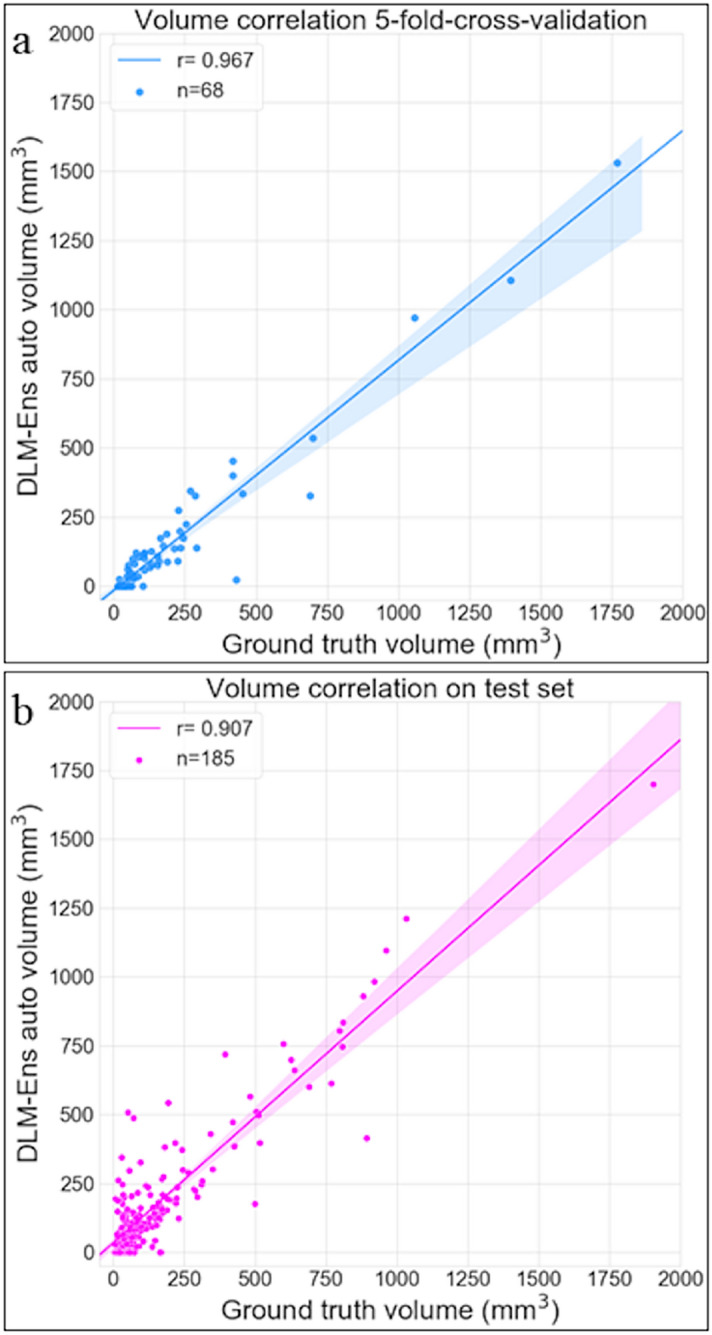
Aneurysm volume correlation plots per patient between manually defined reference standard and the automatically obtained segmentations for validation (a) and test set (b) of DLM-Ens using Pearson correlation (r).

Volume correlation plots between manual and automatic segmentations of training and test set using the DLM are presented in Fig. 4.

#### Overall sensitivity in relation to aneurysm volume

Overall, the DLM achieved a sensitivity of 82% (DSC 0.75, precision 54%) and FPs/scan of 0.81. For aneurysms > 30 mm^3^ (n = 171), sensitivity was 87% (P = 0.23, median DSC 0.80, precision 65%) with FPs/scan of 0.42 (P < .001). Considering aneurysms with volumes of > 50 mm^3^ (n = 124) and > 100 mm^3^ (n = 73), corresponding sensitivities of 90% (P =.05, median DSC 0.84, precision 71%) and 96% (P = .004, median DSC 0.87, precision 73%) were noted. Table 4 provides detailed results.

**Table 4:** Sensitivity, median dice similarity coefficient (DSC), precision, F1 score, and average false positive (FP)/scan of the DLM-Ens in the combined test cohort in relation to aneurysm volume.

	All aneurysms	> 30 mm^3^	> 50 mm^3^	> 100 mm^3^	P value
Sensitivity (detected/missed)	82% (178/37)	87% (148/23)	90% (112/12)	96% (70/3)	.015
DSC (median)	0.75	0.80	0.84	0.87	
Precision (detected/FP)	54% (178/149)	65% (148/81)	71% (112/45)	73% (70/26)	< .001
F1 score	0.66	0.75	0.80	0.84	.019
FP/scan (FP/number of scans)	0.81 (149/185)	0.42 (81/185)	0.24 (45/185)	0.14 (26/185)	< .001

#### Overall sensitivity in relation to aneurysm location

Pairwise comparison indicated a correlation between detection sensitivity and parent artery location. The sensitivity to detect middle cerebral artery (MCA) aneurysms (88%) was significantly higher than for internal carotid artery (ICA) aneurysms (70%, P = .02). Likewise, anterior cerebral artery (ACA) aneurysms (84%) and posterior circulation aneurysms (87%) were associated with higher detection sensitivity than ICA aneurysms. However, these differences did not reach statistical significance. When comparing detection rates of anterior (ICA, ACA, and MCA) with posterior circulation, no statistical significance was found (P = .07).

#### Overall sensitivity in relation to Fisher grade

Detection sensitivity for aneurysms was not significantly impeded by the presence of intracerebral or intraventricular hemorrhage (Fisher ≤ 3: sensitivity 80%, FP/scan 0.74 vs. Fisher 4: sensitivity 85% (P = .33), FP/scan 0.85 (P = .49)).

**Figure 5:**
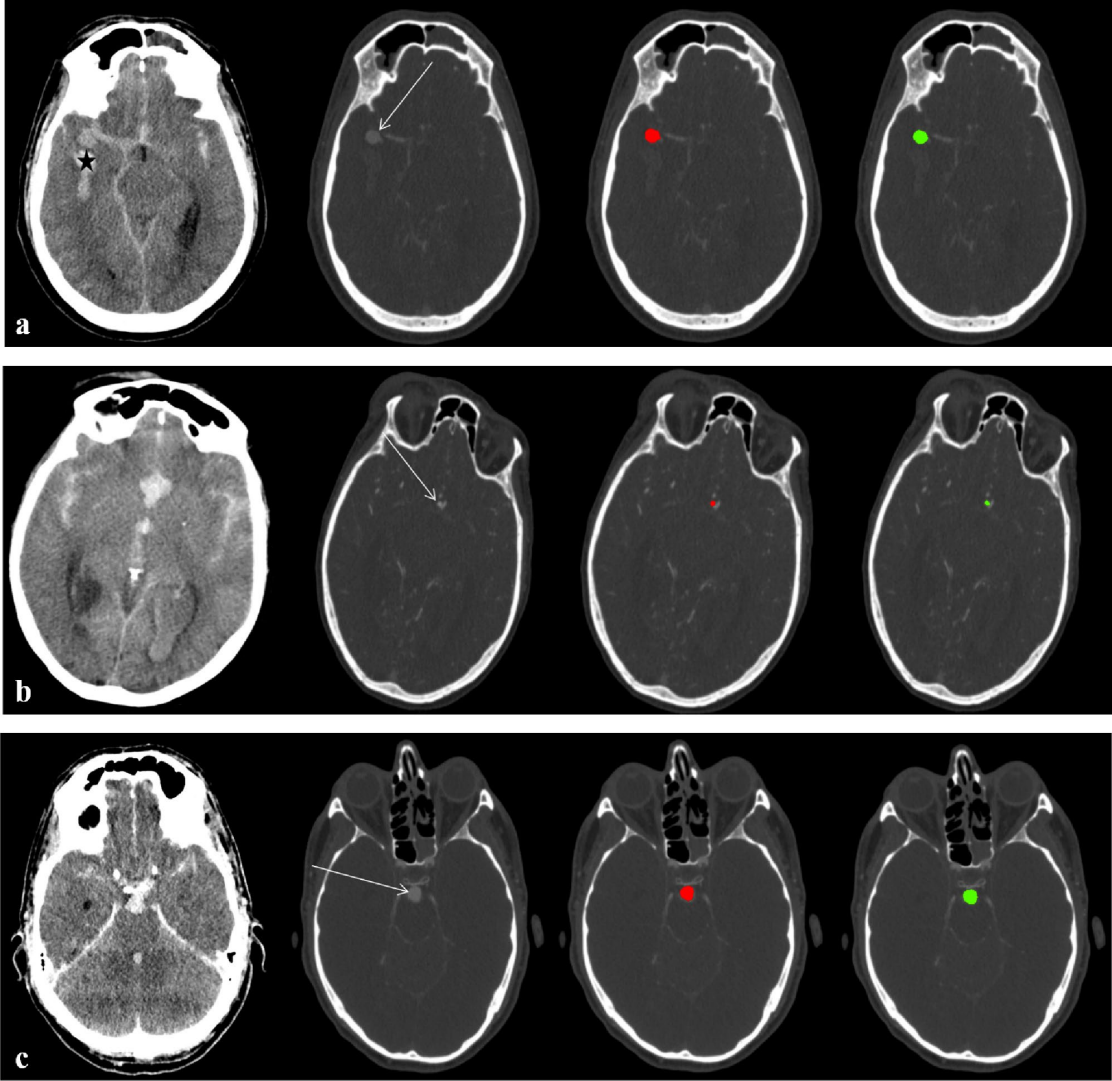
(**a**) Fifty-one-year-old male with Fisher 4 bleeding (* = intraventricular bleeding on axial unenhanced CT) and an aneurysm of the right middle cerebral artery (arrow) on axial CTA source images. The DLM-Ens (green) detects and segments the aneurysm (volume based on manual segmentation: 424.7 mm^3^) with high overlap (DSC of 0.94) compared to manual segmentations (red). (**b**) Sixty-three-year-old female with aSAH on axial unenhanced CT and an anterior communicating artery aneurysm (arrow) on axial CTA source images. Albeit being of small size (volume based on manual segmentation: 25.5 mm^3^), the DLM-Ens (green) detects and segments (DSC of 0.72) the aneurysm with high overlap compared to manual segmentations (red). (**c**) Fifty-one-year-old male with aSAH on axial unenhanced CT and a large basilar tip aneurysm (arrow; volume based on manual segmentation 419.6 mm^3^) on axial CTA source images. Compared to manual segmentations (red), the DLM-Ens (green) provides accurate detection and segmentation (DSC of 0.90).

**Figure 6:**
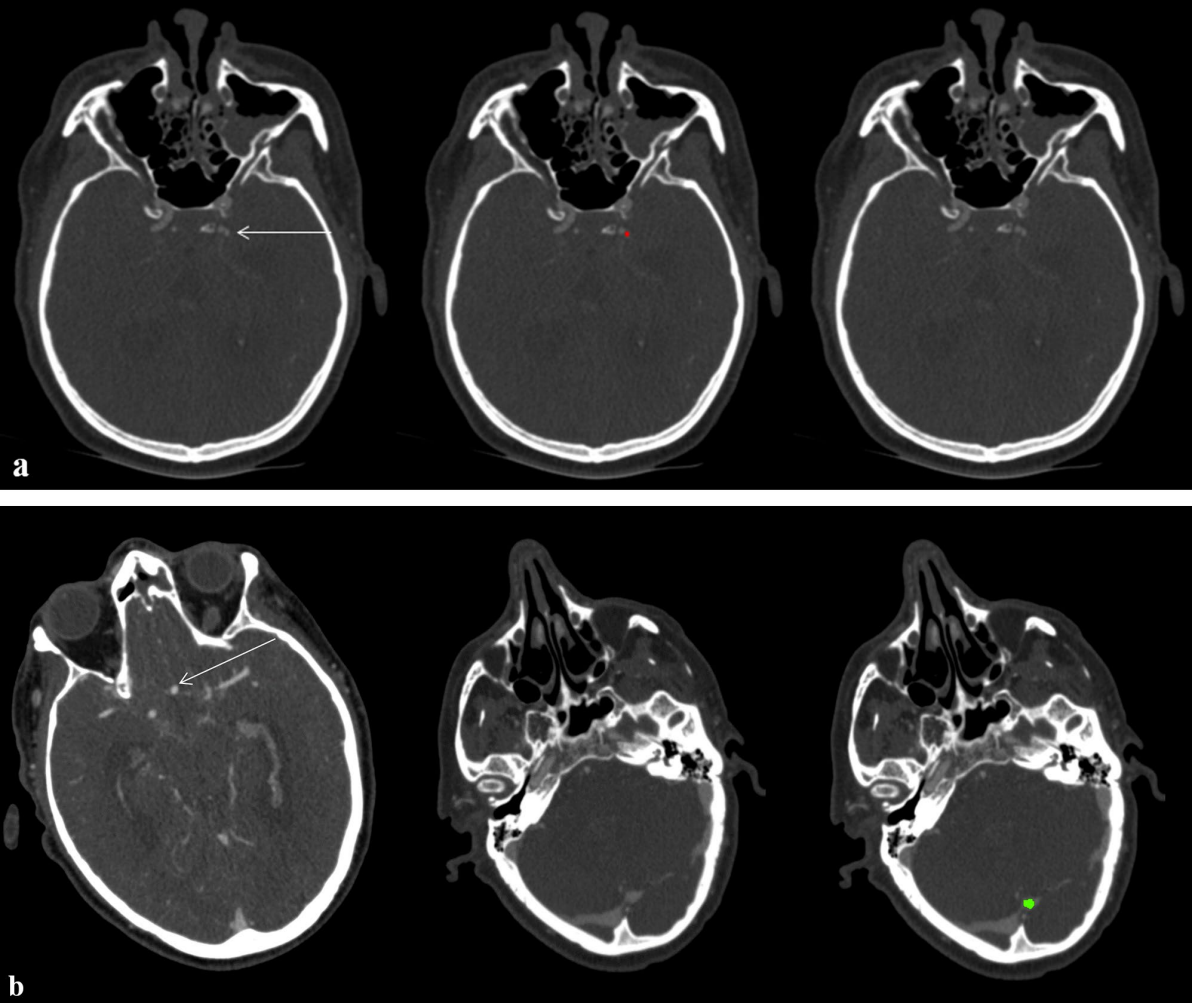
(**a**) Axial CTA source images of a 64-year-old male with a small aneurysm (volume based on manual segmentation: 21.8 mm^3^) of the left internal carotid artery (arrow, red: manual segmentations) being missed by the DLM-Ens. (**b**) Axial CTA source images of a 65-year-old male with an anterior communicating artery aneurysm (arrow), which was accurately segmented and detected by the DLM. However, a false positive finding of the DLM-Ens (in green) at the superior sagittal sinus/transverse sinus was detected.

Exemplary images on the test set regarding detected and missed aneurysms as well as FP findings using the DLM are shown in Figs. 5 and 6.

## Discussion

In the present study, we developed and trained a DLM for automatic detection and segmentation of intracranial aneurysms on CTA in patients with aSAH and evaluated its performance on an independent test set. As a major finding of the study, the DLM achieves a detection rate of 87% for aneurysms > 30 mm^3^ with high segmentation performance and less than one FP finding per scan.

Previous studies have investigated DLMs for detection of UIA providing detection rates between 83 and 95% on TOF-MRA^17,18^ and CTA^10^, respectively. However, these studies reported FPs/scan of 6^18^ and 10^17^ or did not report them^10^, which questions their usefulness in clinical routine. The present study is the first to evaluate the performance of a DLM for automatic detection and segmentation of aneurysms in patients with aSAH. In the current study, the DLM provided a sensitivity of 87% for aneurysms > 30 mm^3^ (~ mean diameter of 4 mm), which is comparable to aforementioned studies by Ueda et al. (83–95% for UIAs > 3 mm on TOF-MRA)^17^, Park et al. (95% for UIAs > 3 mm on CTA)^10^, and Sichtermann et al. (90–93% for UIAs on TOF-MRA)^18^. For aneurysms > 100 mm^3^ (~ mean diameter of 6 mm), sensitivity of the DLM increased to almost 100%.Since aneurysms > 7 mm bear the highest risk of rupture^24–26^, the proposed DLM enables accurate detection of the clinically most relevant aneurysms in aSAH.

Achieving an overall DSC of 0.75 for aneurysm segmentation and of 0.87 for aneurysms > 100 mm^3^, the proposed DLM yields high segmentation performance (superior to the DLM provided by Sichtermann et al.^18^, who reported a DSC of 0.53) despite the small lesion size. Therefore, the DLM can be of assistance to treating physicians regarding treatment planning, e.g. providing 3D geometrical characterization of aneurysms.

Using ensemble learning of three different DLMs, an average number of FPs of less than one per scan was observed, being lower than in aforementioned studies^17,18^. Concerning detection of larger aneurysms (> 100 mm^3^), FPs were almost zero per scan. For remaining findings, majority of FPs were found in venous vessels or adjacent to bone and usually not associated with arterial vessels. Therefore, most of them were easily identifiable as an incorrect finding.

There was no significant difference between the anterior and posterior circulation regarding aneurysm detection. However, ICA aneurysms showed the lowest detection rates with a significant difference compared to the MCA. This discrepancy is most likely due to presence of calcified plaques, dilatation and elongation of the vessel due to hypertension, complex anatomy of aneurysms, and, in particular, proximity to the skull^27^. In future, additional training methods identifying bone geometry might help the DLM to better distinguish bone from adjacent vessels and aneurysms.

Furthermore, we evaluated whether the presence of aSAH would impede the sensitivity of the DLM. Remarkably, the algorithm performed independently of the Fisher grade with additional presence of parenchymal or intraventricular hemorrhage neither decreasing detection rates nor increasing FPs/scan. Therefore, the DLM provides high feasibility and detection sensitivity even in severe aSAH with potential hydrocephalus or midline shift possibly leading to an overall complex image of the brain^28^.

CTA-based detection of intracranial aneurysms can be time consuming and challenging and shows a large variability among physicians, especially for small aneurysms. They present a highly variable interrater agreement depending on various factors, e.g., localization and subspecialty training, hence resulting in a lower detection rate than larger aneurysms even for experienced clinicians^13,14^. In this study, we aimed to overcome these limitations by establishing a robust reference standard based on review of CTA scans and reports by three readers as well as availability of DSA in the majority of patients to find aneurysms potentially not been described in the initial CTA report.

As proof of concept, the results of this study indicate that deep learning is able to provide sufficient detection of aneurysms in aSAH, especially of larger ones, which are bearing the highest risk of rupture^24–26^. With an overall detection rate of 82%, the DLM enables detection rates comparable to a human reader (e.g., sensitivity 83% for UIAs > 3 mm in the study by Park et al.^10^). In this context, the DLM may provide support to physicians that lack concentration due to fatigue or lack of training. This is important in the setting of aSAH if the treating physician detects one aneurysm, preferably the one causing the bleeding, but potentially misses a second aneurysm caused by decreased concentration due to the “satisfaction of search” phenomenon^16,29,30^. Nevertheless, the DLM should be further improved to achieve 100% detection sensitivity, which is required to guarantee patient safety in aSAH.

## Limitations

Besides its retrospective design, our study has a few limitations. Although scans from five different CT scanners were included in this study, 91% were acquired using the iCT, hence the true performance of the DLM on CTA images acquired on different scanners besides the iCT is unknown and the evaluation of generalizability is limited. This limitation should be addressed in future studies. Being a single-center study, only two scans were included from referring institutions, therefore a multi-center study investigating the performance of the DLM on other CT scanners using different protocols should be conducted. Further, we did not include previously treated aneurysms; hence, the detection performance in these patients (with additional artifacts potentially impeding detection) still needs to be investigated.

## Conclusions

In conclusion, we developed a DLM able to provide sufficient detection of aneurysms in aSAH with almost 100% sensitivity for aneurysms > 100 mm^3^ (~ mean diameter of 6 mm). Furthermore, high volumetric correlation to human segmentations and a low number of FPs/scan were obtained. Confounders such as cerebral circulation and bleeding severity did not significantly affect the performance of the DLM. Additional training is required to increase sensitivity for smaller aneurysms; however, the DLM may already be of assistance to treating physicians by providing automated detection of aneurysms in aSAH.

## Abbreviations

ACA: Anterior cerebral artery
aSAH: Aneurysmal subarachnoid hemorrhage
CNN: Convolutional neural network
CTA: CT-angiography
DSA: Digital subtraction angiography
DSC: Dice similarity coefficient
DLM: Deep learning model
FP: False positive
FN: False negative
ICA: Internal carotid artery
ISD: IntelliSpace Discovery
MCA: Middle cerebral artery
TOF-MRA: Time-of-flight magnetic resonance angiography
UIA: Unruptured intracranial aneurysm
RIA: Ruptured intracranial aneurysm
STAPLE: Simultaneous truth and performance level estimation
